# Evolutionary Diversification of Banded Tube-Dwelling Anemones (Cnidaria; Ceriantharia; *Isarachnanthus*) in the Atlantic Ocean

**DOI:** 10.1371/journal.pone.0041091

**Published:** 2012-07-16

**Authors:** Sergio N. Stampar, Maximiliano M. Maronna, Mark J. A. Vermeij, Fabio L. d. Silveira, André C. Morandini

**Affiliations:** 1 Departamento de Zoologia, Instituto de Biociências, Universidade de São Paulo, São Paulo, Brazil; 2 Departamento de Genética e Biologia Evolutiva, Instituto de Biociências, Universidade de São Paulo, São Paulo, Brazil; 3 Carmabi Foundation, Willemstad, Curaçao, Netherlands Antilles; 4 Aquatic Microbiology, Institute for Biodiversity and Ecosystem Dynamics, University of Amsterdam, Amsterdam, The Netherlands; American Museum of Natural History, United States of America

## Abstract

The use of molecular data for species delimitation in Anthozoa is still a very delicate issue. This is probably due to the low genetic variation found among the molecular markers (primarily mitochondrial) commonly used for Anthozoa. Ceriantharia is an anthozoan group that has not been tested for genetic divergence at the species level. Recently, all three Atlantic species described for the genus *Isarachnanthus* of Atlantic Ocean, were deemed synonyms based on morphological simmilarities of only one species: *Isarachnanthus maderensis*. Here, we aimed to verify whether genetic relationships (using COI, 16S, ITS1 and ITS2 molecular markers) confirmed morphological affinities among members of *Isarachnanthus* from different regions across the Atlantic Ocean. Results from four DNA markers were completely congruent and revealed that two different species exist in the Atlantic Ocean. The low identification success and substantial overlap between intra and interspecific COI distances render the Anthozoa unsuitable for DNA barcoding, which is not true for Ceriantharia. In addition, genetic divergence within and between Ceriantharia species is more similar to that found in Medusozoa (Hydrozoa and Scyphozoa) than Anthozoa and Porifera that have divergence rates similar to typical metazoans. The two genetic species could also be separated based on micromorphological characteristics of their cnidomes. Using a specimen of *Isarachnanthus bandanensis* from Pacific Ocean as an outgroup, it was possible to estimate the minimum date of divergence between the clades. The cladogenesis event that formed the species of the Atlantic Ocean is estimated to have occured around 8.5 million years ago (Miocene) and several possible speciation scenarios are discussed.

## Introduction

In addition to morphological and/or ecological descriptions, genetic diversity within geographically separated populations can provide useful information to identify Cnidarian species e.g. [1]; [2]; [3]; [4]. Genetic studies on cnidarian populations reveals not only cryptic species, but also provides information on the processes and scenarios that could have led to speciation in the marine environment e.g., [5]; [6]; [7] and illustrate how past oceanographic and geological events have shaped current distributional patterns of marine biodiversity e.g., [8]; [9]. The existence of cryptic species and unknown dynamics on geological timescales often hamper such phylogeographic studies.

Isarachnanthus Carlgren, 1924 is a genus of tube forming anemones within the order Ceriantharia [10] that extend their tentacles only during the night [8]. Among anthozoans, Ceriantharia is a clade whose taxonomic status is currently debated; it is sometimes placed among clades of the Hexacorallia [11] and sometimes deemed an ancestral clade of all other Anthozoa clades [12]. Unlike many classic anthozoan taxa, Ceriantharia species have larvae with a long planktonic life stage which led earlier researchers to confuse them with jellyfishes, i.e., Medusozoa [13]. This long pelagic life stage or pelagic larval duration (PLD) provides Isarachnanthus species with a high dispersal potential with possible consequences for cladogenesis within this taxon [14]. The length of species’ PLD has been related to speciation patterns in other marine organisms [15]; [16]; [17].

Currently, the genus Isarachnanthus globally consists of three species [18]; [10], [19]. For the Pacific region two species were described and accepted as valid, Isarachnanthus bandanensis (Carlgren, 1924) and Isarachnanthus panamensis (Carlgren, 1924). Three species were originally described for the Atlantic Ocean, Isarachnanthus maderensis (Johnson, 1861) from Madeira Island (32°38′50″N 16°50′51″W), Isarachnanthus nocturnus (den Hartog, 1977) from Curaçao (Caribbean Sea –12°07′49″N 68°58′07″W) and Isarachnanthus cruzi (Brito, 1986) from the Canary Islands (27°51′2″N 15°23′43″W). However, a morphological comparison of these three Atlantic species recently indicated no apparent differences between specimens collected from the Caribbean Sea, Canary Islands and Madeira Island [19]. Consequently, these Atlantic species were considered synonyms of Isarachnanthus maderensis.

The status of Ceriantharia species, as well as species statuses in other cnidarian taxa, is often debated; a situation that even worsened when molecular studies indicated that cryptic species are common in Cnidaria e.g. [1]. Nevertheless, molecular markers have been successfully used to define and delimitate species, especially in genera in which species share similar morphologies or when convergent evolution has caused geographically separated species to develop similar morphological characteristics e.g. [20]; [21]. The most widely used molecular marker Cytochrome Oxidase I (DNA barcoding) generally shows an extremely low rate of divergence between closely related anthozoan species of the same genus [22]: see more in [23].

Based on a morphological and genetic comparison of Isarachnanthus species collected from Atlantic and Pacific Ocean we aimed, for the first time, to review the status of the genus Isarachnanthus in Atlantic Ocean and discuss the most likely evolutionary scenarios underlying speciation in this genus as a latter input, the new genetic data would enhance the discussion about the systematic position of the group.

## Results

### Systematics

#### Morphological differences among species

**Figure 1:**
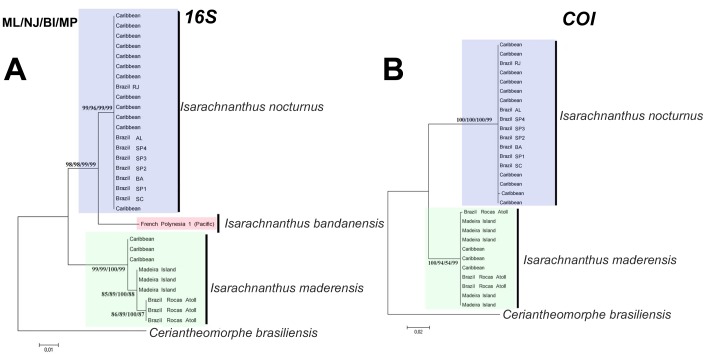
Phylogenetic reconstructions (Maximum likelihood) of the analyzed specimens of *Isarachnanthus* using the mitochondrial markers 16s and COI. Number on the branches represent the estimated values of maximum likelihood (ML), maximum parsimony (MP), neighbor joining (NJ) and Bayesian inference (BI), respectively.

**Figure 2:**
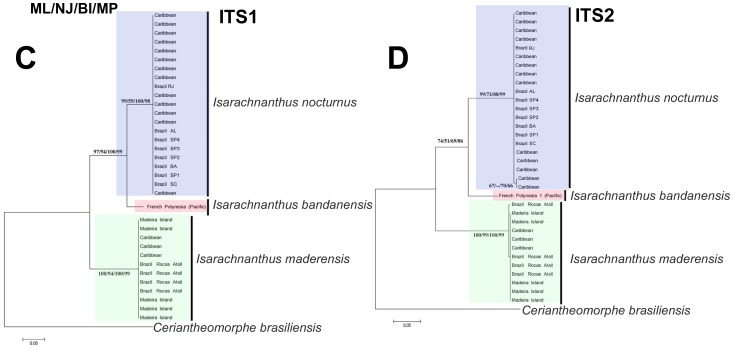
Phylogenetic reconstructions (Maximum likelihood) of the analyzed specimens of *Isarachnanthus* using the nuclear markers ITS1 and ITS2. Number on the branches represent the estimated values of maximum likelihood (ML), maximum parsimony (MP), neighbor joining (NJ) and Bayesian inference (BI), respectively.

There are no external or internal macromorphological differences between Isarachnanthus specimens collected from the Caribbean, Brazilian coast and Northeast Atlantic (Madeira) as previously reported by [19]. Tentacles and mesenterial arrangement, coloration pattern, siphonoglyph and mesenteries form are similar among these specimens. The cnidome contained more than thirty kinds of cnidae, and only four types could be used to significantly distinguish between presumed species (Table 1– types with *) and for which size (expressed as length and width) could be used to significantly distinguish among species (Table 2). Furthermore, six different types of cnidae (Table 1– types with #) were unique for certain species, five types were exclusively found in Isarachnanthus maderensis and one type in Isarachnanthus nocturnus. The micromorphological analysis of the species’ cnidomes allows a partial distinction of specimens from Brazil, Caribbean Sea and Madeira Island, however it is not obvious without molecular data (Table 1). The length of several cnida types of specimens from different areas and different species overlapped and provided no resolution to distinguish among species (see Figures 1 and 2). The specimen of Isarachnanthus bandanensis from French Polynesia (Pacific) showed four series of labial tentacles, whereas the two Caribbean species I. maderensis and I. nocturnus have only one series. We had no access to specimens of I. panamensis from the Pacific, so this species is not further considered in this study.

**Table 1:** Cnidome of the studied specimens of *Isarachnanthus*.

	*Isarachnanthus maderensis*(Madeira Island)	*Isarachnanthus maderensis*(Rocas Atoll, Brazil)	*Isarachnanthus nocturnus*(Curaçao)	*Isarachnanthus nocturnus*(São Sebastião, Brazil)
**MARGINAL TENTACLES**				
Atrich	54.36 **(51–57)×**7.26 **(6–7.26)**	51.48 **(49.2–54.6)×**8.82 **(8.4–9.6)**	65.43 **(61.8–73.8)×**9.72 **(9–10.8)**	48.12 **(46.8–49.2)×**8.34 **(7.2–9)**
Microb. B-mastigophore 1 *	14.22 **(12–16.2)×**3.54 **(3–3.6)**	15.3 **(13.2–18)×**5.04 **(4.8–5.4)**	18.66 **(18–21)×**4.62 **(3.6–4.8)**	19.02 **(18–21)×**6.12 **(6–6.6)**
Microb. B-mastigophore 2	104.88 **(100.8–114)×**25.5 **(24–28.8)**	101.97 **(96–108)×**26.52 **(21–30)**	112.02 **(107.4–116.4)×26.79 (25.2–27.6)**	**–**
Microb. B-mastigophore 2	53.64 **(50.4–59.4)×**7.38 **(7.2–7.8)**	49.56 **(43.8–54.6)×**5.82 **(5.4–6)**	68.34 **(63–72)×**6.66 **(6–7.8)**	61.5 **(57–66)×**9.06 **(8.4–9.6)**
Microb. P-mastigophore	37.98 **(36–42)×**4.44 **(4.2–5.4)**	35.94 **(31.2–42)×**4.2 **(4.2)**	44.58 **(43.2–46.8)×**5.64 **(5.4–6)**	37.92 **(36–39) ×** 6.18 **(5.4–7.8)**
Microb. P-mastigophore	–	–	–	21.72 **(21–22.8) ×** 3.6 **(3.6)**
**LABIAL TENTACLES**				
Atrich	48.6 **(43.2–54) ×** 7.14 **(6.6–7.8)**	46.38 **(40.8–52.8) ×** 8.7 **(7.2–9)**	60.33 **(52.8–64.8) ×** 8.4 **(7.2–9)**	32.34 **(28.8–36) ×** 6.18 **(6–6.6)**
Microb. B-mastigophore 1 *	37.08 **(36–38.4) ×** 5.52 **(5.4–6)**	49.98 **(42–54) ×** 7.2 **(6–8.4)**	18.18 **(16.8–21) ×** 4.62 **(3.6–4.8)**	13.44 **(12.6–15.6) ×** 3.6 **(3.6)**
Microb. B-mastigophore 2	75.09 **(72–79.2) ×** 15.27 **(13.8–16.2)**	100.08 **(92.4–108) ×** 24.18 **(21–27.6)**	85.71 **(81–91.2) ×** 15.45 **(15–16.2)**	62.94 **(57–66) ×** 9 **(8.4–9.6)**
Microb. B-mastigophore 2	53.58 **(48–59.4) ×** 8.52 **(7.2–9.6)**	74.28 **(69–78.6) ×** 14.64 **(14.4–15)**	69.96 **(67.8–75) ×** 11.04 **(10.2–11.4)**	27.9 **(27–30) ×** 6.48 **(6–6.6)**
Microb. P-mastigophore	33.66 **(30–38.4) ×** 5.64 **(5.4–6)**	32.82 **(30–35.4) ×** 4.38 **(4.2–4.8)**	41.13 **(36–46.8) ×** 5.4 **(5.4)**	22.5 **(20.4–24) ×** 3.42 **(3–3.6)**
**STOMODEUM**				
Atrich	48.12 **(43.2–52.8) ×** 7.08 **(6.6–7.8)**	59.88 **(51–69.6) ×** 9.54 **(9–9.6)**	77.46 **(66–85.2) ×** 9.12 **(7.2–9.6)**	41.76 **(39–47.4) ×** 7.32 **(6.6–8.4)**
Microb. B-mastigophore 1	38.16 **(36–40.8) ×** 5.76 **(5.4–6.6)**	21.48 **(20.4–22.8) ×** 4.2 **(4.2)**	44.82 **(41.4–48.6) ×** 6.54 **(6–6.6)**	22.02 **(21–23.4) ×** 6.84 **(6.6–7.2)**
Microb. B-mastigophore 2	74.52 **(73.4–78) ×** 15.3 **(15–15.6)**	67.02 **(63–75) ×** 14.94 **(14.4–15.6)**	81.24 **(78–87) ×** 16.5 **(15.6–18)**	59.7 **(57–63) ×** 9.66 **(9–10.8)**
Microb. B-mastigophore 2 #	53.64 **(49.2–59.4) ×** 8.52 **(7.2–9.6)**	50.52 **(48–54) ×** 9.18 **(8.4–9.6)**	**–**	**–**
Microb. P-mastigophore	33.84 **(31.2–38.4) ×** 5.58 **(4.8–6)**	36.72 **(33–39.6) ×** 7.08 **(6.6–7.2)**	63.96 **(57–70.8) ×** 11.64 **(10.2–12.6)**	23.4 **(21.6–24.6) ×** 3.6 **(3.6)**
**COLUMN**				
Atrich a	–	46.02 **(43.2–51.6) ×** 15.84 **(14.4–18)**	62.88 **(57–69) ×** 8.7 **(8.4–9)**	42.9 **(36–48) ×** 7.86 **(6.6–8.4)**
Atrich b *	37.86 **(36–40.8) ×** 6.18 **(5.4–7.2)**	31.44 **(28.8–31.44) ×** 6.96 **(6.6–7.2)**	28.92 **(21–33) ×** 6.75 **(6–7.8)**	22.62 **(21–30) ×** 5.7 **(5.4–6)**
Pticocysts	44.97 **(36–54) ×** 11.25 **(9.6–12.6)**	67.38 **(63–69.6) ×** 14.16 **(13.2–15)**	85.95 **(81.6–93) ×** 18.3 **(17.4–19.8)**	43.14 **(36–48) ×** 12.18 **(9.6–13.2)**
Microb. B-mastigophore 1	22.8 **(20.4–25.2) ×** 5.82 **(5.4–6.6)**	22.26 **(18–24) ×** 5.82 **(5.4–6)**	30.18 **(27.6–33.6) ×** 8.13 **(7.2–9)**	21.84 **(19.2–25.2) ×** 6.06 **(6–6.6)**
Microb. B-mastigophore 2	89.04 **(84–96) ×** 22.02 **(19.2–24)**	89.46 **(86.4–96) ×** 14.04 **(13.2–15)**	71.28 **(69–75.6) ×** 9.24 **(9–9.6)**	48.3 **(39–54) ×** 8.88 **(7.8–9)**
Microb. B-mastigophore 2 #	78.42 **(72–81) ×** 14.16 **(12–15.6)**	66.72 **(57–72.6) ×** 11.76 **(10.8–12.6)**	**–**	**–**
Microb. P-mastigophore #	22.8 **(20.4–25.2) ×** 5.82 **(5.4–6.6)**	23.1 **(22.2–24) ×**7.5 **(7.2–7.8)**	**–**	**–**
**M-MESENTERIES (DOUBLE CORD)**				
Atrich	36.24 **(31.2–42) ×** 6.06 **(6–6.6)**	49.32 **(45.6–55.2) ×**9.06 **(8.4–9.6)**	63.6 **(57–72) ×**9.69 **(9.6–10.2)**	42.78 **(39–45.6) ×**8.4 **(7.8–9)**
Microb. B-mastigophore 1 #	–	–	23.28 **(19.2–26.4) ×**5.94 **(5.4–6)**	22.62 **(21–24.6) ×**8.28 **(7.2–9.6)**
Microb. B-mastigophore 2 #	87.06 **(83.4–92.4) ×**23.82 **(21.6–27)**	69 **(66–71.4) ×**14.4 **(13.8–15)**	**–**	**–**
Microb. B-mastigophore 2	77.94 **(72–84) ×**14.04 **(12.6–16.2)**	58.98 **(57–61.2) ×**9.66 **(9–10.2)**	75.84 **(72–78.6) ×**15.6 **(15–17.4)**	46.14 **(42–48.6) ×**8.94 **(8.4–9)**
Microb. B-mastigophore 2	18.6 **(16.8–21)×**6.9 **(6.6–7.8)**	**–**	**–**	**–**
Microb. P-mastigophore	40.14 **(37.2–42.6)×**6.18 **(6–6.6)**	32.24 **(28.8–34.8) ×**4.86 **(4.8–5.4)**	37.86 **(35.4–39.6) ×**6 **(6)**	39.54 **(36–45) ×**6.36 **(6–7.2)**
**B-MESENTERIES (SIMPLE CORD)**				
Microb. B-mastigophore 1	20.88 **(18.6–24) ×**6.36 **(6–6.6)**	36.06 **(34.8–37.8) ×**5.64 **(5.4–6)**	19.47 **(17.4–22.8) ×**6 **(6)**	16.26 **(15–17.4) ×**6.6 **(6.6)**
Microb. B-mastigophore 1	–	19.98 **(18–21.6) ×**2.94 **(2.4–3)**	21.36 **(20.4–23.4) ×**5.43 **(5.4–6)**	22.98 **(21.6–24) ×**4.8 **(4.8)**
Microb. B-mastigophore 2 #	18.12 **(16.2–19.8) ×**6.3 **(6–7.6)**	31.02 **(30–33.6) ×**4.5 **(4.2–4.8)**	**–**	**–**
Acontiods				
Atrich *	41.1 **(39–46.8) ×**7.8 **(7.2–9)**	40.38 **(39–42) ×**7.92 **(7.2–8.4)**	51.24 **(48–57) ×**9 **(8.4–9.6)**	53.64 **(49.2–55.2) ×**8.1 **(7.8–8.4)**
Microb. B-mastigophore 1	18.24 **(14.4–21) ×**4.62 **(3.6–5.4)**	15.72 **(15–16.8) ×**4.98 **(4.8–5.4)**	16.8 **(15–19.2) ×**3 **(3)**	18.6 **(18–20.4) ×**3.4 **(3–3.6)**
Microb. B-mastigophore 1	19.56 **(18–21) ×**7.44 **(7.2–8.4)**	24.96 **(24–27.6) ×**3.66 **(3.6–4.2)**	23.52 **(21–24.6) ×**5.58 **(4.8–6)**	23.34 **(19.8–25.2) ×**5.7 **(4.8–6)**
Microb. B-mastigophore 2	86.52 **(81–92.4) ×**25.5 **(24–27.6)**	88.38 **(84–94.8) ×**15.72 **(15.6–16.2)**	90.51 **(84–96) ×**22.23 **(18–25.8)**	89.1 **(84–96) ×**20.82 **(18–24)**

# and * highlights are cnidae types with differences between species. The sizes are expressed as: mean of length (maximum and minimum) × mean of width (maximum and minimum) in µm.

**Table 2:** Mann-Whitney test comparing the measurements (length and width) of cnidae (* Table 1) between the species *Isarachnanthus maderensis* and *Isarachnanthus nocturnus*. Since p-value smaller than 0,05 infers that samples are different.

Cnidae	Body part	Measure	P-value
Microbasic	Marginal tentacle	length	<0.0001
B-mastigophore 1		width	<0.0001
	Labial tentacle	length	<0.0001
		width	<0.0001
Atrich	Column	length	<0.0001
		width	0.006
	Acontiods	length	<0.0001
		width	<0.0001

Since *p-value* smaller than 0,05 infers that samples are different.

### Molecular Classification

**Figure 3:**
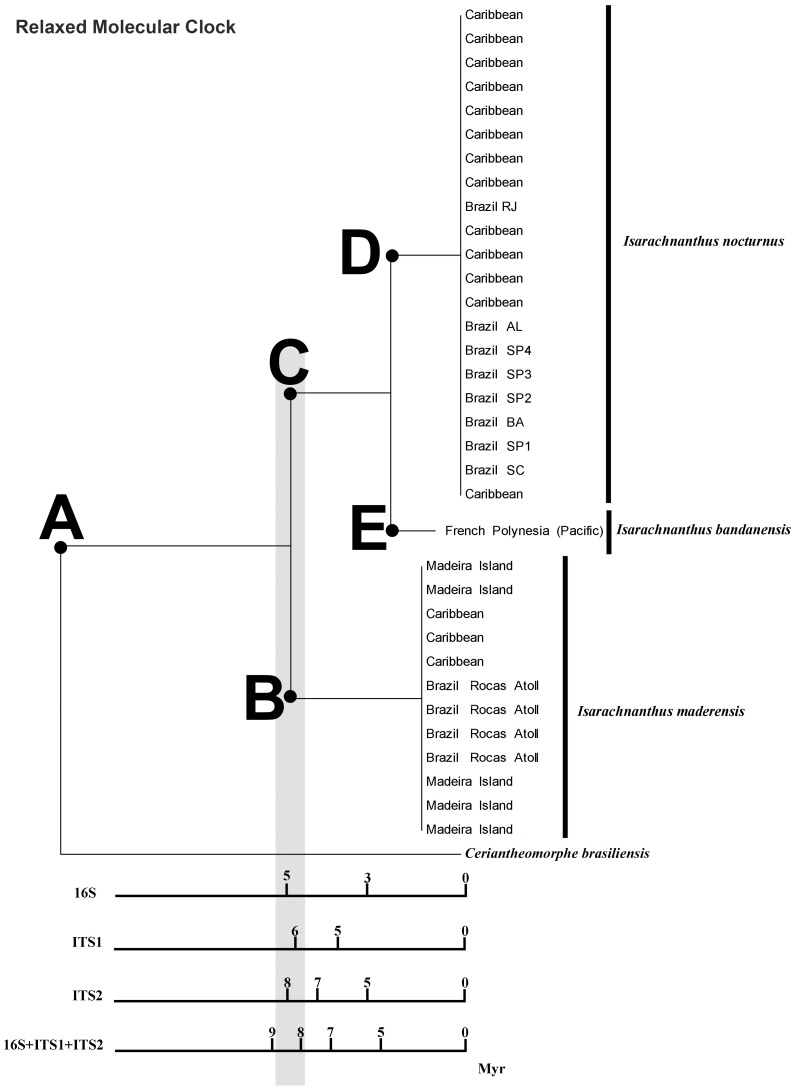
Analysis of maximum likelihood with estimation of relaxed molecular clock dating the node *Isarachnanthus bandanensis* X *Isarachnanthus nocturnus* with the closure of Isthmus of Panama. Note that each bar represents a marker used in the analysis and the last with three concatenated markers. Myr – Millions of years. A – The ancestral species of *I. bandanensis* + *I. maderensis* + *I. nocturnus*; B – *Isarachnanthus maderensis*; C – The ancestral species of *I. bandanensis* + *I. nocturnus*; D – *Isarachnanthus nocturnus* and E – *Isarachnanthus bandanensis*.

Maximum Likelihood, Neighbor Joining, Bayesian Inference and Maximum Parsimony analyzes of the genus Isarachnanthus from Atlantic waters based on the genetic data from molecular markers 16S, COI, ITS 1 and 2 revealed two distinct but cohesive clades with high support values (Figures 1 and 2). One clade (named Isarachnanthus nocturnus - blue) is confined to the west coast of the Atlantic (Brazilian coastal waters and Caribbean Sea). The other clade (named Isarachnanthus maderensis - green) occurs on both sides of the Atlantic (Madeira Island, Rocas Atoll and Caribbean Sea). Therefore, two distinctive clades occur in the Atlantic Ocean that overlap in the Caribbean region. A sample from French Polynesia (Isarachnanthus bandanensis) was used to estimate the minimum time of divergence between the studied clades (Figure 3).

In contrast to the commonly observed low interspecific genetic variation found in other Anthozoa [24], Ceriantharia show significant genetic separation among closely related, i.e., morphologically similar species using standard genetic markers. The estimated divergence between I. maderensis and I. nocturnus is 18% for ITS-1; 27% for ITS-2, 9% for COI and 6% for 16S. Variation within each species for the same markers was always less than 1%.

### Molecular Clock and Historical Context

The speciation event that separated I. nocturnus and I. bandanensis, occurred later than the speciation event that separated the I. maderensis and the I. nocturnus + I. bandanensis clade (Figure 3). The separation of the Atlantic I. maderensis and Caribbean I. nocturnus + Pacific I. bandanensis clade therefore likely coincided with geological events that caused gene-flow between the two regions to stop, such as, for example, the closure of the Isthmus of Panama that occurred ±4.5 mya (see material and methods section).

The isolated analysis of mitochondrial marker, 16S, showed an unlike differentiation time between the species than nuclear markers ITS1 and ITS2 (see material and methods section). An analysis with concatenated markers was therefore applied to present a more conservative pattern. Assuming that the concatenated analysis represents all nucleotide diversity of molecular markers used in this section see more in [25], we decided to use this result as our working hypothesis. Based on the results of concatenated analyzes, the speciation period between Isarachnanthus maderensis and I. nocturnus + I. bandanensis occured 9 to 8 mya (Figure 3).

The rate of divergence of the COI in Cnidaria is estimated at 1% per million year [26]. When this rate was applied to the data from this study, we find a speciation period around 9 mya between Isarachnanthus maderensis and I. nocturnus + I. bandanensis. That is, using an independent manner of the dating also shows the same pattern/scale found in the above tests.

## Discussion

### Systematics

Based on our micromorphological (cnidae) and molecular results, we conclude that there are two different *Isarachnanthus* species in the Atlantic Ocean. However, the distribution of both species overlaps in the Caribbean region. Variations in the cnida size is a very complicated character to be used as an conclusive diagnostic characteristic to identify species in some Anthozoa see [27] including *Isarachnanthus* (this study), though the presence and absence of some cnida types could potentially be used as a taxonomic character in Ceriantharia see Table 2 in [28].

The genetic divergence found among the studied species corresponds with that observed in certain Medusozoa species [29], but differs considerably from patterns generally observed in Anthozoa [30]. Up to now the analysis of genetic divergence found among species/genera of several Anthozoa groups (mainly using the molecular markers COI and 16S) are hardly successful to discern among presumed lineages and morphologically defined species. The data we obtained from Ceriantharia’s specimens analyzed in our study differs from this pattern commonly observed in anthozoans, mostly due to the larger genetic distances between species in the different taxa. Anthozoans are considered one of the metazoans with lowest rate of mitochondrial evolution, *i.e.*, more than 100 times slower than in most other marine invertebrates [31]. These results call our attention to ressurrect the discussion on the systematic position of Ceriantharia as an independent lineage from traditional Anthozoans groups. Slow mitochondrial divergence were considered as a shared characteristic of basal metazoan and cnidarian groups [32] and rates of mitochondrial genome evolution were used to argue for cnidarian relationships and patterns of evolution. For example, mitochondrial genetic divergence was presented as evidence to suggest that the Medusozoa clade is a cnidarian “derived group”. New medusozoan mitochondrial genomes had been published [33] [2] and their results sustain these general evolutionary profile, even considering the mitochondrial linear genomic arquitecture in medusozoans [34]. Previous results present Ceriantharia possibly as sister group of all other anthozoans [12]; [35] and our observations support this hypothetic phylogenetic position.

While the morphological differences between *Isarachnanthus maderensis* and *Isarachnanthus nocturnus* were subtle and only observable after detailed microscopic analyses, a molecular approach successfully supported the distinction of two Atlantic species. This suggests that ecological traits (*e.g.* physiology, life cycle, substrate preference) rather than morphological characteristics could drive speciation in this genus. Based on our results and [13], *Isarachnanthus nocturnus* shows a large latitudinal distribution in comparison with *Isarachnanthus maderensis*. The environmental conditions presented in the coast of southeastern Brazil to northern Argentina are different (*e.g.* temperature, water turbidity) from those found in the areas of the Caribbean Islands and Northeast Atlantic (Madeira) [36]; [37]. From this perspective, *I. nocturnus* occurs across a larger gradient of environmental conditions than *I. maderensis* thus potentially reflecting its wider tolerance or adaptability to varying environmental conditions.

### Distribution of the Species

The species *Isarachnanthus maderensis* has a distribution restricted to the part of the North Atlantic that is subjected to the Subtropical Gyre (Gulf Stream, North Equatorial Current): Madeira Island (also the type locality) and the Caribbean Sea, but also to Rocas Atoll (off northeast Brazil) which is under the influence of the South Equatorial Current [38]. The South Equatorial Current meets the Brazil Current (around latitude 6° and 10° S) [39], but no individuals of *Isarachnanthus maderensis* were found in Brazilian coastal waters. The area under the influence of the Brazil Current was only inhabited by individuals of *Isarachnanthus nocturnus*. Therefore, two possibilities exist that explain the occurrence of *Isarachnanthus maderensis* in Rocas Atoll. The first is that the Rocas Atoll sometimes comes under the influence of the North Equatorial Current as evidenced by the fact that European garbage was found in the region of the Rocas Atoll [40]. The second explanation involves physiological or ecological restrictions to the species, which prevent it to inhabit the coastal regions of Brazil.

### Tempo and Spatial Dynamics of Ceriantharia Speciation (Molecular Clock)

**Figure 4:**
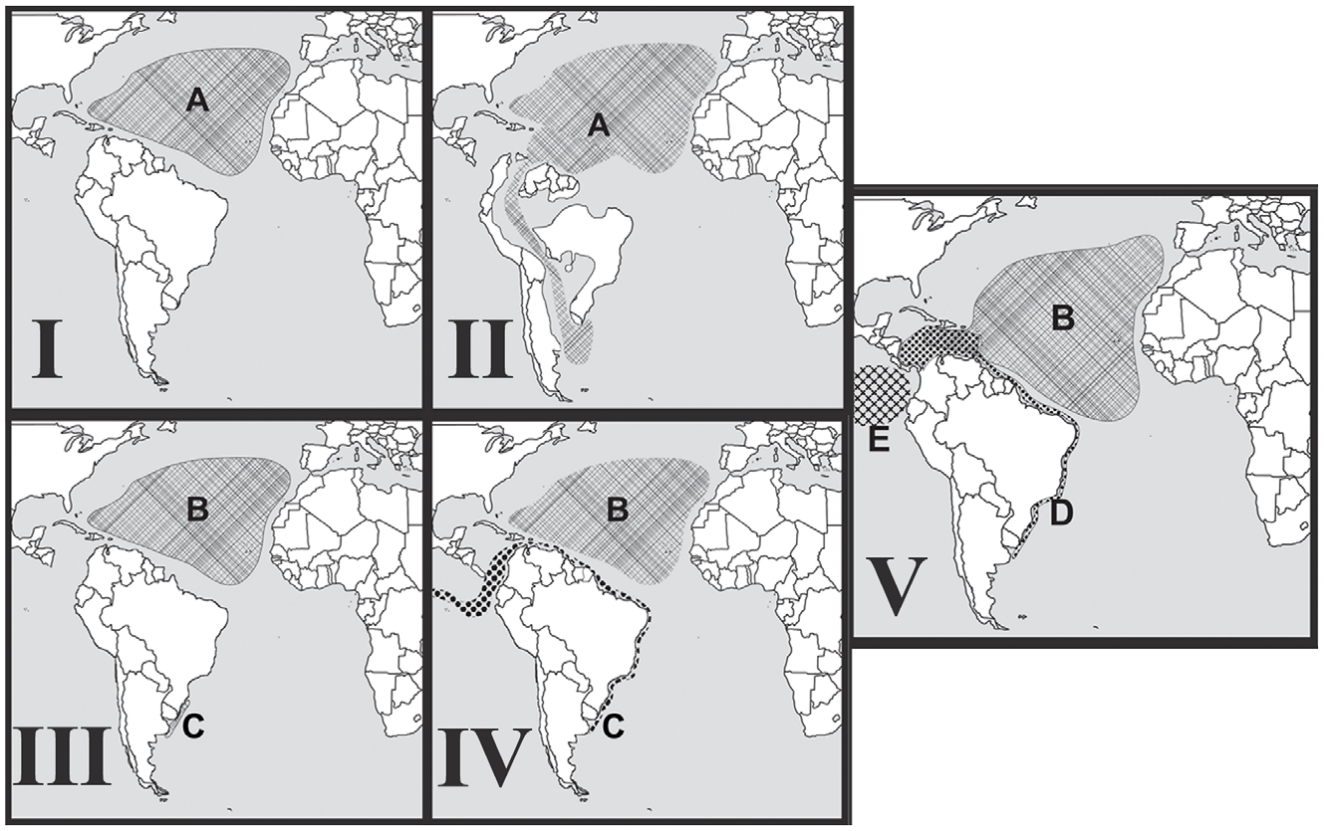
Possible scenario to explain the recent distribution of *Isarachnanthus* species and the patterns of species differentiation. Scenario 1– The hypothesis of peripatric and allopatric speciation through the intracontinental seaway. I – scenario around 16 myr before today; II – scenario around 11 myr before today; III – scenario around 8 myr before today; IV – scenario around 6 myr before today and V – currently scenario. A – The ancestral species of *I. bandanensis* + *I. maderensis* + *I. nocturnus*; B – *Isarachnanthus maderensis*; C – The ancestral species of *I. bandanensis* + *I. nocturnus*; D – *Isarachnanthus nocturnus* and E – *Isarachnanthus bandanensis*.

The minimum age for the divergence between the *Isarachnanthus maderensis* and *Isarachnanthus nocturnus* + *Isarachnanthus bandanensis* clades is estimated to have occurred around 8.5 million years ago, *i.e.*, the late Miocene (Tortonian Period). For this time period two possible speciation scenarios can be proposed. The first scenario would have occurred by subsequent peripatric and allopatric speciation events (Figure 4). During the Tortonian period an internal seaway formed between the Caribbean Sea and the Southwest Atlantic [41]–[42] (Figure 4 - I). The existence of this internal sea connection in the middle of the South American continent during the Late or Middle Miocene (Figure 4) has been accepted by many authors [41]–[42]. Some authors estimated that the Atlantic Ocean moved over the South America continent about 11 to 9 million years ago which is supported by both geological [43] and biological/paleontological data [44], but see: [45]. The seaway could have allowed the ancestral species to enter the southwest Atlantic (Figure 4 - II). After the seaway closed, one population became restricted to what is now northern Argentina, Uruguay and southern Brazil (Figure 4 - III). The other population remained in the tropical Atlantic. These populations remained isolated for a long time, probably during all Messinian period [46] after which the southern population moved back to the equatorial zone by longshore current or drift see more in [47] and [48] (Figure 4 - IV). However this possibility could only have taken place after the great ice age during the late Tortonian and Messinian [49]. During this period, a large reduction in sea level caused a major change in ocean currents, including a large ascending current of the southern Argentina to present Caribbean Sea [50]. Apparently this distribution expansion occurred just before the closing of the Isthmus of Panama which segregated the coastal South American species, resulting in the species currently recognized, *Isarachnanthus nocturnus* (Atlantic) and *Isarachnanthus bandanensis* (Pacific) (Figure 4 - V). Here, we assumed that *Isarachnanthus maderensis* originated during the first speciation event (Figure 4 - III) and was already present in the Caribbean Sea region during the second speciation event (Figure 4 - V).

**Figure 5:**
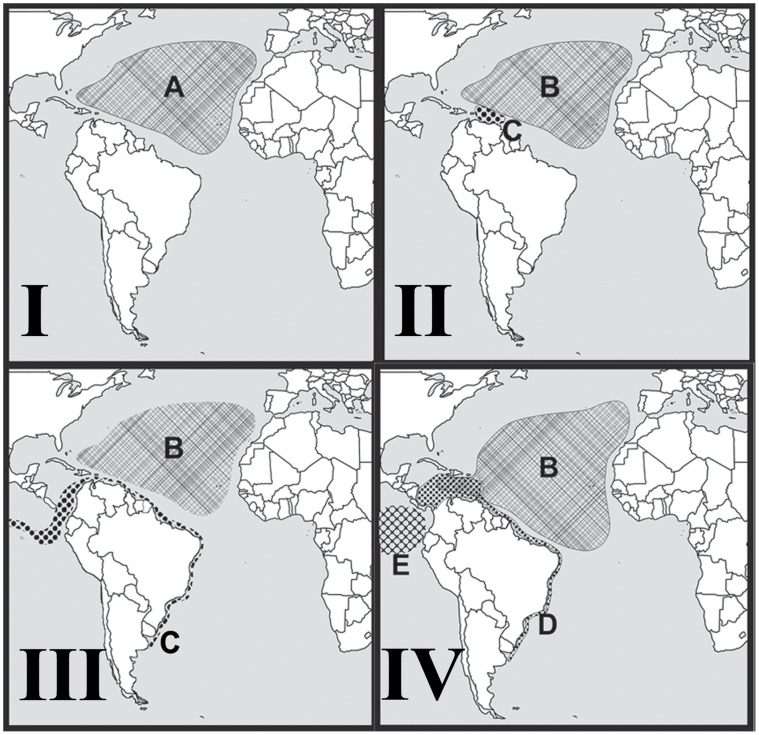
Possible scenario to explain the recent distribution of *Isarachnanthus* species and the patterns of species differentiation. Scenario 2 - The hypothesis of sympatric and allopatric speciation. I – scenario around 16 myr before today; II – scenario around 11–8 myr before today; III – scenario around 6 myr before today; IV – currently scenario. A – The ancestral species of *I. bandanensis* + *I. maderensis* + *I. nocturnus*; B – *Isarachnanthus maderensis*; C – The ancestral species of *I. bandanensis* + *I. nocturnus*; D – *Isarachnanthus nocturnus* and E – *Isarachnanthus bandanensis*.

The second scenario could have occurred by subsequent events of allopatric and sympatric speciation (Figure 5). In this hypothesis the first speciation event occurred through sympatric speciation [51] (Figure 5 - II). One of the species formed during this speciation may have been able to expand its distribution to coastal environments in the southwest Atlantic Ocean and the Pacific Ocean (Figure 5 - III). This pattern of two species was then broken up during the closure of the Isthmus of Panama, resulting in allopatric speciation and the distribution of species as they were found in this study (Figure 5 - IV).

The two scenarios mentioned above are both plausible and difficult to prove. Marine species can originate through allopatric divergence, where new species arise from geographically isolated populations of one ancestral species [52]; [53]. The first scenario is interesting given that the proposed period during which speciation occurred coincides with a debated geological event [28]. Meanwhile explaining the absence of the ancestral species (A) in the Pacific Ocean region is difficult. The obstacle of the second scenario is to understand the first speciation event, sympatric (A→B+C). Sympatric/ecological speciation has become more accepted in recent decades, however in general it remains difficult to conclusively prove that sister species have speciated through sympatric processes alone [54]–[55].

The use of DNA barcode (COI) in Anthozoa was tested in Ceriantharia. Although not appropriate in other groups *e.g*. [24] this tool is fully useful for identification of species of Ceriantharia, mainly by the genetic distance observed. The mitochondrial DNA barcode “approach” is often deemed unsuitable to study Anthozoa due to the extremely low rate of mitochondrial divergence between species. Our study advocates the use of morphological characters in association with molecular data from both genomes, in an integrative approach, to better understand and explain the diversity patterns observed nowadays.

## Materials and Methods

### Specimens Sampling

Specimens were sampled by SCUBA using a shovel in the areas listed in Table 3. Each animal was directly preserved in Ethanol 95%. The molecular analyses were based on 25 individuals of *Isarachnanthus nocturnus*, 13 of *Isarachnanthus maderensis* and one of *Isarachnanthus bandanensis*. For the morphological study we used the same individuals of the molecular analyses with the addition of 10 specimens of *I. nocturnus* and 6 specimens of *I. maderensis*. Specimens from the type localities of each previously described species were used in the molecular and morphological analysis.

**Table 3:** Taxa included in this study with sampling area of the analyzed material and GENBANK number of each molecular marker.

Sampling area	Lat/Long	Species*	16S	COI	ITS1/ITS2
Brazil/REBio Arvoredo – SC	–27.27/−48.36	*I. nocturnus*	JX125673	JX128315	JX125636
Brazil/São SebastiãoChannel – SP	–23.82/−45.42	*I. nocturnus*	JX125669/JX125675/JX125698/JF915194/JF915192	JX128316/JX128318JF915196-97/JX128341-42	JX125637/JX125639-41/JX125632-33
Brazil/Forno Beach – RJ	–22.96/−42.01	*I. nocturnus*	JX125684	JX128327	JX125650
Brazil/Boa Viagem Beach – BA	–12.94/−38.50	*I. nocturnus*	JX125674	JX128317	JX125638
Brazil/Frances Beach – AL	–9.71/−35.79	*I. nocturnus*	JX125676	JX128319	JX125642
Brazil/Rocas Atoll – RN	–3.86/−33.80	*I. maderensis*	JX125685-87	JX128328-30	JX125651-54
Curaçao – Piscadera Bay	12.12/−68.96	*I. nocturnus*	JX125677-78/81/83JX125688-97	JX128320-21/24/26	JX125643-44/47/49JX125658-67
Curaçao – Piscadera Bay	12.12/−68.96	*I. maderensis*	JX125679-80/82	JX128334-40	JX125645-46/48
Portugal – Madeira Island	32.63/−16.84	*I. maderensis*	JX125670-JX125672	JX128322-23/25JX128331-33	JX125634-36/JX125655-57
French Polynesia –Moorea Island	–17.47/−149.81	*I.bandanensis*	JX125699	–	JX125668
Brazil/Florianopolis – SC	–27.43/−48.45	*Ceriantheomorphe* *brasiliensis*	JF915193	JF915195	JX138232

* Defined *a posteriori* – AL – Alagoas, BA – Bahia, RJ – Rio de Janeiro, RN – Rio Grande do Norte, SC – Santa Catarina and SP – São Paulo.

All necessary permits were obtained for the described field studies (sampling). Samples that occurred inside Environmental Protected Areas of Brazil were covered by license SISBIO 10508.

### Data Collection

#### Molecular study

DNA was extracted using InstaGene (Bio-Rad) from single tentacles removed from the specimens. Genes were amplified using the PCR technique, then PCR products purified with AMPure® kit (Agencourt®). The PCR primers CB1 (forward - TCGACTGTTTACCAAAAACATA) and CB2 (reverse - ACGGAATGAACTCAAATCATGTAAG) [56] were used to amplify part of the 16S gene (expected fragment of 435 to 681 bp), LCO1490 (GGTCAACAAATCATAAAGATATTGG) and HCO2198 (TAAACTTCAGGGTGACCAAAAAATCA) [57] to amplify part of the COI gene (expected fragment of 670 to 804 bp) (mitochondrial markers, ribosomal and protein coding genes respectively). Primers jfITS-5f (GGTTTCCGTAGGTGAACCTGCGGAAGGATC) [1], and CAS28sB1d (TTCTTTTCCTCCSCTTAYTRATATGCTTAA) [58] were used to amplify the target fragment of the nuclear ribosomal unit, including the complete Internal Transcriber Spacer 1 (expected fragment of 121 to 129 bp), the 5.8S Ribosomal Subunit and the Internal Transcriber Spacer 2 (expected fragment of 203 to 227 bp). Purified PCR’s products were made ready to sequencing using the BigDye® Terminator v3.1 kit (Applied Biosystems), with the same primers and temperature conditions of the PCR’s reactions. The sequencing procedure was carried out on an ABI PRISM®3100 genetic analyzer (Hitachi).

### Data Analysis

#### DNA Analysis

Sequences were assembled and edited (removing ambiguous base calls and primer sequences) using Geneious™ 5.4.4 [59]. The alignment in every molecular marker were made using MUSCLE in default parameters [60]. New sequences were submitted to GenBank (Table 3). Kimura’s two-parameter model of base substitution was used to calculate genetic distances in MEGA5 software [61]. The maximum likelihood phylogenetic analysis was conducted via RAxML (500 replicates) (random accelerated maximum likelihood analysis) with general time reversible model and gamma rate heterogeneity (GTR+GAMMA) [62]. Neighbor joining and maximum parsimony analysis were conducted via Mega 5.5 [60]. In the case of maximum parsimony, trees were obtained by the search of trees algorithm of CNI [63] with support estimation assessed with 500 bootstrap replicates and Kimura’s two-parameter model. The NJ tree was obtained by bootstrap method (500 replicates) with uniform rates, Kimura’s two-parameter model and gaps as complete deletion. Finally, Bayesian inference were performed via MrBayes 3.2 [64] implemented in Geneious™ 5.4.4 [59] with GTR+GAMMA Model (chain length  = 1100000, subsampling frequency  = 200, burn-in length  = 100000 and random seed 27265) and other parameters in default.

To estimate the divergence time between clades and time of speciation of the sister species, we used the software Mega 5.5 [61], BEAST 1.6.2, BEAUTi 1.6.2 and Tree Annotator 1.6.2 [65]. In this test the tree obtained via maximum likelihood analysis (RAxML) was dated to the node known (closure of the Isthmus of Panama −4.5 million years on conservative estimates) [66]; [67]; [68]. The NEXUS file was obtained in BEAUTi 1.6.2 with general time reversible (GTR) model and Gamma distributed rate variation across sites. The Gamma distribution was assumed with an error of ±1 million years. Tree prior was defined as Yule Process. The Markov chain Monte Carlo (MCMC) parameters were defined in 1 million cycles to length chain and MCMC samples were printed to the screen and logged to files every 1000 cycles. Two independent replicates were performed to check for the convergence of the estimates. The programs TreeAnnotator v1.6.2 and FigTree v1.3.1 were used to summarize the posterior tree distribution and to visualize the annotated maximum clade credibility (MCC) tree, respectively.

### Morphological Analysis

The anatomical study of the polyps and cnidome were based on criteria defined by several authors [69]; [70]; [10]. The classification of studied cnidae followed [10]. The cnidome was based on thirty measures of undischarged cnidae of specimens preserved in 4% formaldehyde solution. Measurements were taken from each cnida type for each body region of specimens from the four distinct geographic areas (Brazil coast, Rocas Atoll, Caribbean Sea and Madeira Island). The specimens were dissected through the ventral side with a cut using surgical scalpels (carbon steel), then the opened body was fixed using acupuncture needles. The cnidome was analyzed under a Nikon Eclipse 80i microscope. All parts of the body were analyzed separately so that any contamination would be avoided. The cnidae that showed no overlap in size were tested via Mann-Whitney test in order to check the consistency of divergence between species.
