# Correction: Evolutionary Diversification of Banded Tube-Dwelling Anemones (Cnidaria; Ceriantharia; *Isarachnanthus*) in the Atlantic Ocean

**DOI:** 10.1371/journal.pone.0109481

**Published:** 2014-09-23

**Authors:** The PLOS ONE Staff

There are errors in Table 3 of the published article. The correct Table 3 can be viewed here.

**Table 3:** Taxa included in this study with sampling area of the analyzed material and GENBANK number of each molecular marker.

Sampling area	Lat/Long	Species*	16S	COI	ITS1/ITS2
**Brazil/REBio Arvoredo - SC**	-27.27/-48.36	*I. nocturnus*	JX125673	JX128315	JX125636
**Brazil/São Sebastião Channel - SP**	-23.82/-45.42	*I. nocturnus*	JX125669/JX125675/JX125698/JF915194/JF915192	JX128316/JX128318JF915196-97/JX128341-42	JX125637/JX125639-41/JX125632-33
**Brazil/Forno Beach – RJ**	-22.96/-42.01	*I. nocturnus*	JX125684	JX128327	JX125650
**Brazil/Boa Viagem Beach – BA**	-12.94/-38.50	*I. nocturnus*	JX125674	JX128317	JX125638
**Brazil/Frances Beach – AL**	-9.71/-35.79	*I. nocturnus*	JX125676	JX128319	JX125642
**Brazil/Rocas Atoll – RN**	-3.86/-33.80	*I. maderensis*	JX125685-87	JX128328-30	JX125651-54
**Curaçao – Piscadera Bay**	12.12/-68.96	*I. nocturnus*	JX125677-78/81/83JX125688-97	JX128320-21/24/26JX128334-40	JX125643-44/47/49JX125658-67
**Curaçao – Piscadera Bay**	12.12/-68.96	*I. maderensis*	JX125679-80/82	JX128322 - 23/25	JX125645-46/48
**Portugal - Madeira Island**	32.63/-16.84	*I. maderensis*	JX125670-JX125672	JX128331-33	JX125634-35/JX125655-57
**French Polynesia – Moorea Island**	-17.47/-149.81	*I.bandanensis*	JX125699	—	JX125668
**Brazil/Florianopolis - SC**	-27.43/-48.45	*Ceriantheomorphe brasiliensis*	JF915193	JF915195	JX138232

* Defined *a posteriori* – AL – Alagoas, BA – Bahia, RJ – Rio de Janeiro, RN – Rio Grande do Norte, SC – Santa Catarina and SP – São Paulo.
